# Ancient founder mutation in *RUBCN*: a second unrelated family confirms Salih ataxia (SCAR15)

**DOI:** 10.1186/s12883-020-01761-w

**Published:** 2020-05-25

**Authors:** Mohammed Z. Seidahmed, Muddathir H. Hamad, Albandary AlBakheet, Salah A. Elmalik, Abdulmajeed AlDrees, Jumanah Al-Sufayan, Ibrahim Alorainy, Ibrahim M. Ghozzi, Dilek Colak, Mustafa A. Salih, Namik Kaya

**Affiliations:** 1 Neonatology Unit, Department of Pediatrics, Security Forces Hospital, Riyadh, 11481 Saudi Arabia; 2 Division of Pediatric Neurology, Department of Pediatrics, College of Medicine, King Saud University, Riyadh, Saudi Arabia; 3 Department of Genetics, King Faisal Specialist Hospital and Research Centre, MBC: 03, P.O. Box 3354, Riyadh, 11211 Kingdom of Saudi Arabia; 4 Department of Physiology, College of Medicine, King Saud University, Riyadh, Saudi Arabia; 5 Department of Radiology and Diagnostic Imaging, College of Medicine, King Saud University, Riyadh, Saudi Arabia; 6 Department of Internal Medicine, Division of Neurology, Security Forces Hospital, Riyadh, Saudi Arabia; 7 Department of Biostatistics, Epidemiology, and Scientific Computing, King Faisal Specialist Hospital and Research Center, Riyadh, Saudi Arabia

**Keywords:** Salih ataxia, SCAR15, RUBCN, Founder mutation

## Abstract

**Background:**

Homozygous frameshift mutation in RUBCN (KIAA0226), known to result in endolysosomal machinery defects, has previously been reported in a single Saudi family with autosomal recessive spinocerebellar ataxia (Salih ataxia, SCAR15, OMIM # 615705). The present report describes the clinical, neurophysiologic, neuroimaging, and genetic findings in a second unrelated Saudi family with two affected children harboring identical homozygous frameshift mutation in the gene. It also explores and documents an ancient founder cerebellar ataxia mutation in the Arabian Peninsula.

**Case presentation:**

The present family has two affected males (aged 6.5 and 17 years) with unsteady gait apparent since learning to walk at 2.5 and 3 years, respectively. The younger patient showed gait ataxia and normal reflexes. The older patient had saccadic eye movement, dysarthria, mild upper and lower limb and gait ataxia (on tandem walking), and enhanced reflexes in the lower limbs. Cognitive abilities were mildly impaired in the younger sibling (IQ 67) and borderline in the older patient (IQ 72). Nerve conduction studies were normal in both patients. MRI was normal at 2.5 years in the younger sibling. Brain MRI showed normal cerebellar volume and folia in the older sibling at the age of 6 years, and revealed minimal superior vermian atrophy at the age of 16 years. Autozygome and exome analysis showed both affected have previously reported homoallelic mutation in RUBCN (NM_014687:exon18:c.2624delC:p.A875fs), whereas the parents are carriers. Autozygosity mapping focused on smallest haplotype on chromosome 3 and mutation age analysis revealed the mutation occurred approximately 1550 years ago spanning about 62 generations.

**Conclusions:**

Our findings validate the slowly progressive phenotype of Salih ataxia (SCAR15, OMIM # 615705) by an additional family. Haplotype sharing attests to a common founder, an ancient RUBCN mutation in the Arab population.

## Background

Homozygous frameshift mutation in RUBCN (Synonym: KIAA0226), known to result in endolysosomal machinery defects, has previously been reported in a single Saudi family with autosomal recessive spinocerebellar ataxia (Salih ataxia, SCAR15, OMIM # 615705) [1, 2]. This consanguineous Saudi Arabian family had 3 sisters with unsteady gait apparent since learning to walk in two and around age 7 years in the third. Two developed epilepsy in infancy which was responsive to treatment and later showed moderate intellectual disability (ID). Two patients had increased reflexes in the lower limbs. Brain MRI showed mild cerebellar atrophy and prominent folia in one patient at age 18 years and was normal at ages 16, 9, and 8 years, respectively.

Recently, the same homozygous frameshift mutation (NM_014687:exon18:c.2624delC:p.A875fs) in RUBCN was identified in a Saudi boy, belonging to an unrelated family with ataxia, who had his DNA analyzed in a joint clinical genomic sequencing analysis project [3]. This multicenter project explored the utility of autozygome analysis for the high throughput confirmation of previously published tentative links to diseases. In the present communication we detail the updated clinical features of the disease in the previously reported proband [3] and report the phenotype and genotype of his older affected 17-year-old brother. We also compare the features of this second Saudi family to that reported in the previous unrelated Saudi family [1]. As well, we also detail the genomic analysis of this second family including the healthy parents (first-degree cousins) and patients, and two normal siblings (a brother and a sister). Considering the similarity of the detected variant in the present family to that previously reported in the Saudi family [1, 2], we explored the possibility of founder effect.

## Case presentation

### Patient 1

**Fig. 1:**
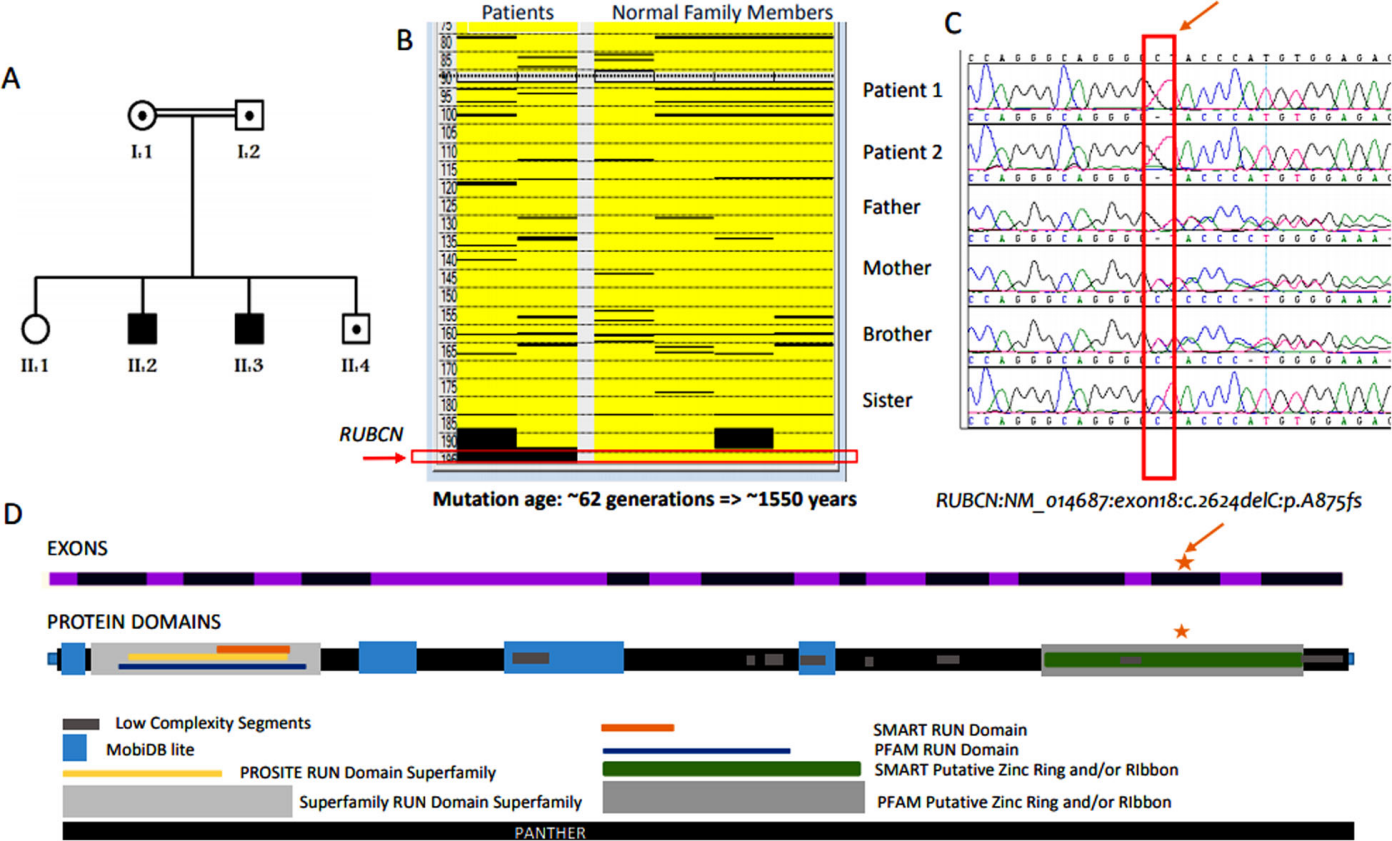
Genetic testing results. **a** Pedigree of the family showing the two affected patients (II.2 and II.3). Another sibling (II.4) is a carrier as well as both parents (I.1 and I.2). **b** AutoSNPa analysis shows runs of homozygosity (ROH) [shown as black blocks] between healthy individuals versus affected individuals in the family. Here, chromosome 3 is displayed (as *RUBCN* is located at the end of q arm). The numbers on the left are the genomic coordinates on the chromosome 3. **c** The ROH block containing *RUBCN* was taken into consideration during the whole exome sequencing (WES) filtering. Based on that, a homozygous variant, a single base deletion of cytosine in 2624th position, (NM_014687:exon18:c.2624delC:p.A875fs) indicated by a red arrow (also shown as in a red box) was detected in *RUBCN* (1c, arrow). **d** The image displays schematic drawing of the exons of the gene and encoded protein domains (arrow). The star shows the position of the variant

The proband (Fig. 1, II.3) is a 6.5- year-old Saudi boy born by emergency cesarean section as preterm (gestational age 33 weeks) to first cousin parents. He had respiratory distress and required oxygen via nasal canula, but settled quickly. He had no dysmorphic features and neurologic and other systems examination revealed normal findings. Following discharge from the neonatal intensive care unit (NICU) and during follow up in clinic, he was found to have delayed developmental milestones. He sat unsupported at 1 year, walked at 30 months and showed unsteadiness since starting to walk. Speech acquisition was delayed till 5 years.

**Fig. 2:**
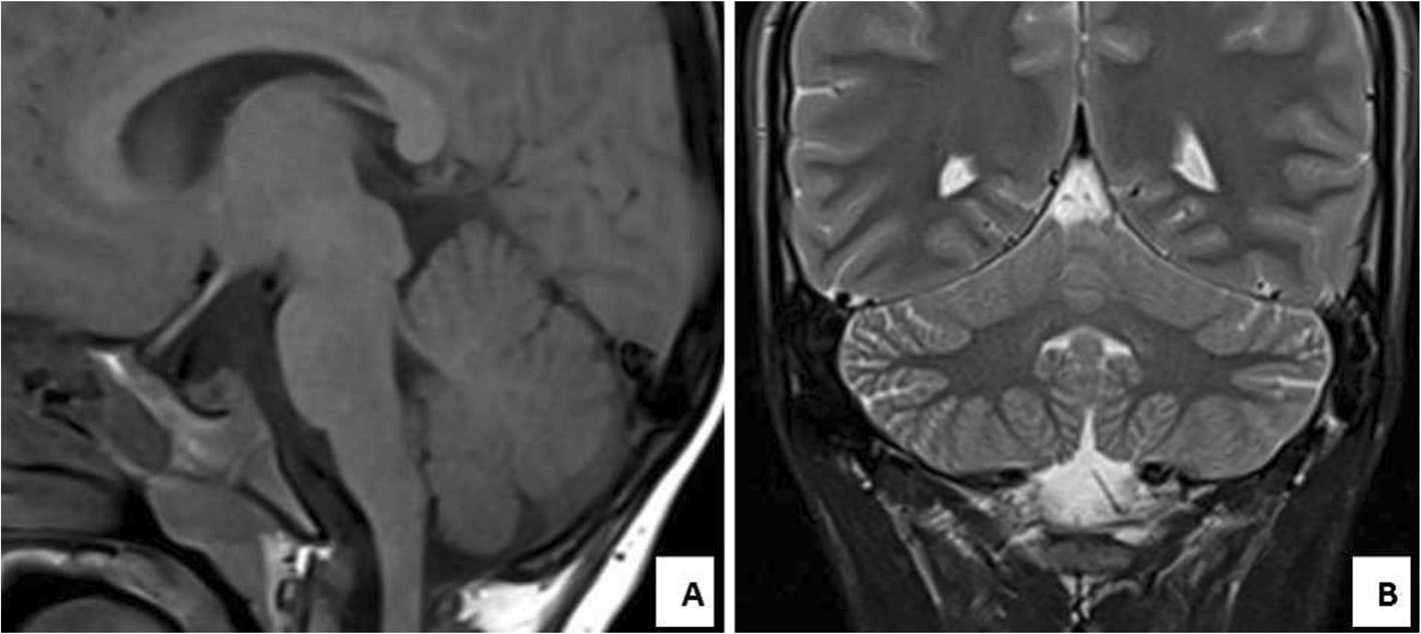
Patient 1 (aged 2.5 years). **a** Sagittal T1 and (**b**) Coronal T2-weighted MR images of the cerebellum showing normal cerebellar volume and folia with no signs of atrophy. The vermis has normal size. The cerebellar gray and white matter are also normal

Examination at the age of 6.5 years showed no craniofacial abnormalities, normal head size (47.5 cm) and normal systemic examination. There was no muscle wasting, fasciculation, ocular apraxia, nystagmus or saccadic pursuit. Muscle tone, power, sensation and deep tendon reflexes were normal. Plantar response was flexor. There were no signs of ataxia in either the upper or lower limbs. IQ testing revealed mildly impaired cognitive abilities (IQ = 67). Laboratory investigations showed normal complete blood count (CBC), electrolytes, creatine kinase (CK), hepatic and renal function tests. Screen for metabolic disorders revealed no abnormalities. Visual evoked potential (VEP) was normal in both eyes, and brain auditory evoked response.

(BAER) showed normal results. Nerve conduction study was essentially normal in the upper and lower limbs. Magnetic resonance imaging (MRI) at the age of 2.5 years (Fig. 2a and b) showed normal cerebellar volume and folia with no signs of atrophy. The vermis had normal size and the cerebellar gray and white matter were also normal.

### Patient 2

The older brother of the proband (Fig. 1, II.2) was evaluated at the age of 17 years. History revealed delayed developmental milestones. He managed to walk at 3 years and showed unsteadiness since starting to walk. Speech acquisition was also delayed till 4 years and remained dysarthric. He also had moderate intellectual disability, detected since early childhood, and was enrolled in special education schooling.

Examination showed no dysmorphic features, normal head size (52.7 cm) and normal systemic examination. There was no muscle wasting or fasciculation, and ocular examination revealed saccadic pursuit. Muscle tone, power, and sensation were normal. Deep tendon reflexes were normal in the upper limbs and enhanced in the lower limbs. Plantar response was flexor. Testing for coordination revealed mild upper and lower limb and gait ataxia (on tandem walking). IQ testing revealed borderline cognitive abilities (IQ = 72).

**Fig. 3:**
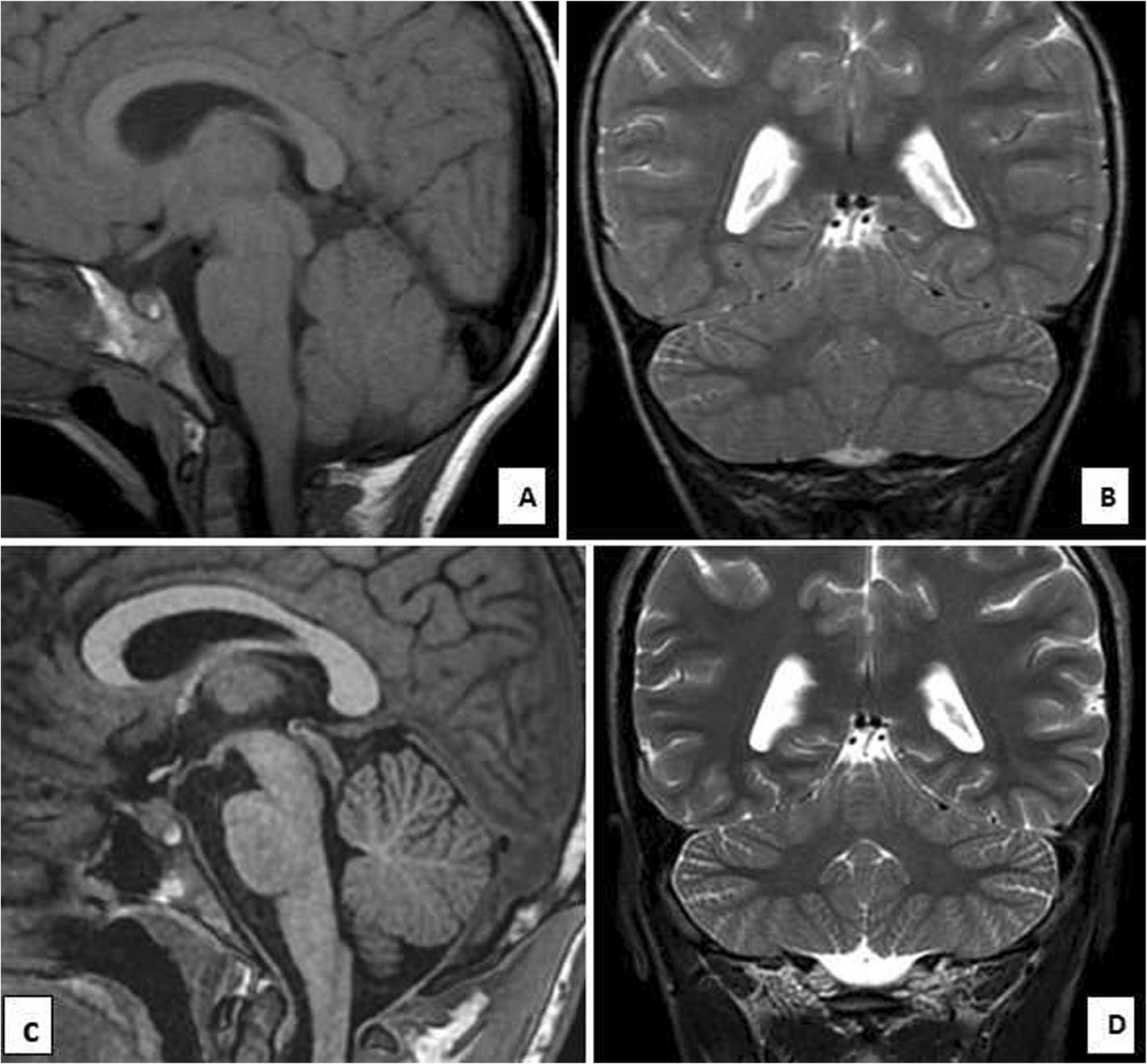
Patient 2. **a** and **b** MR images at the age of 6 years (**a**) Sagittal T1 and (**b**) Coronal T2- weighted MR images of the cerebellum showing normal cerebellar volume and folia with normal vermis and no signs of atrophy. **c** and **d** MR images at the age of 16 years (**c**) Sagittal T1 and (**d**) Coronal T2-weighted MR images of the cerebellum showing minimal superior vermian atrophy. The other parts of the cerebellum show normal volume and folia with no signs of atrophy. The cerebellar gray and white matter are also normal

Visual evoked potential (VEP) was normal in both eyes, and brain auditory evoked response (BAER) revealed increased hearing threshold in left (40 dB) and right (60 dB) ears. Nerve conduction study was normal in the upper and lower limbs. Brain MRI at the age of 6 years (Fig. 3a and b) showed normal cerebellar volume and folia with normal vermis and no signs of atrophy. At the age of 16 years MRI revealed minimal superior vermian atrophy (Fig. 3c and d). The other parts of the cerebellum showed normal volume and folia with no signs of atrophy. The cerebellar gray and white matter were also normal. A summary of the clinical and neuroimaging findings in the present and previously reported Saudi families is depicted in Table 1.

**Table 1:** Clinical and neuroimaging findings in patients with Salih ataxia (autosomal recessive spinocerebellar ataxia-15, SCAR15) due to founder mutation in *RUBCN* gene

	This report		Assoum et al [1]		
Gender ((Pedigree No.)	Patient 1 M	Patient 2 M	Patient 1 F	Patient 2 F	Patient 3 F
Age at assessment	6.5 y	17 y	16 y	19 y	16 y
Initial symptoms	Unsteadiness (at 2.5 y)	Unsteadiness (at 3 y)	Epilepsy (at 7 mo)	Unsteadiness (at 7 y)	Epilepsy (at 7 mo)
Delayed motor development	Yes, walked at 2.5 y	Yes, walked at 3 y	Yes, walked at 42 mo	Yes, walked at 22 mo	Yes, walked at 28 mo
Delayed speech acquisition	Yes, talked at age 5y	Yes, talked at age 4 y	Yes, talked at age > 4 y	Yes, talked at age 3 y	Yes, talked at age 4 y
Cognitive deficit	Yes (IQ = 67)	Yes, attending special school (1Q = 72)	Yes, moderate intellectual disability	None	Yes, moderate intellectual disability
Onset of dysarthria	Since talking	Since talking	Since talking	Since talking	Since talking
Abnormal eye movements	None	Saccadic pursuit	Nystagmus	Saccadic pursuit	Saccadic pursuit
**Cerebellar ataxia**:					
Upper limbs	None	Mild	Mild	Mild	Severe
Lower limbs	None	Mild	Moderate	Moderate	Severe
Gait	None	Mild	Moderate	Moderate	Severe
Dysarthria	Mild	Mild	Moderate	Moderate	Severe
Disability stage^a^	0	1	3	3	3
**Deep tendon reflexes**:					
Upper limbs	Normal	Normal	Diminished	Diminished	Diminished
Lower limbs	Normal	Enhanced	Diminished	Enhanced	Enhanced
Plantar reflex	Flexor	Flexor	Flexor	Flexor	Flexor
BAER (Age when done)	Normal (6.5 y)	Increased hearing threshold in L (40 dB) and R (60 dB) ears (17 y)	Normal in L ear. Increased hearing threshold in R ear [40 dB] (14 y)	Not done	Increased hearing threshold in L (60 dB) and R (40 dB) ears (16 y)
VEP (Age when done)	Normal (6.5 y)	Normal (17 y)	Normal (14 y)	Not done	Bilateral optic pathway involvement [P100 latency = 135.5 msec in L eye and 137 msec in R eye] (16 y)
Motor and sensory NCS (Age when done)	Normal (6.5 y)	Normal (17 y)	Normal (24 y)	Normal (19 y)	Normal (16 y)
MRI brain (Age when done)	Normal (2.5 y)	Normal (6 y), minimal superior vermian atrophy (16y)	Normal (16 y)	Normal (9 y), mild cerebellar atrophy and prominent folia (18 y)	Normal (8 y)

*Abbreviations*: *BAER* Brain auditory evoked responses, *F* Female, *IQ* Intelligence quotient, *L* Left, *M* Male, *mo* months, *MRI* Magnetic resonance imaging, *NCS* Nerve conduction study, *R* Right, *VEP* Visual evoked potentials, *y* years

^a^Disability Stage: 0 = No functional handicap; 1 = No functional handicap but signs at examination; 2 = Mild, able to run, walking unlimited; 3 = Moderate, unable to run, limited walking without aid; 4 = Severe, walking with one stick; 5 = Walking with two sticks; 6 = Unable to walk, requiring wheelchair; 7 = Confined to bed

After clinical evaluation, the DNA samples (from all the family members including patients 1 and 2) were sent out for genetic testing (Fig. 1a). As part of routine diagnostic procedures, the DNA samples were run for WES analysis and filtered according to published protocols [4–8]. Then all the DNA samples were also genotyped using Genechip axiom arrays (Affymetrix Inc.) that consists more than 600,000 SNPs distributed throughout the human genome. Following the genotyping, all SNP calls were transferred to AutoSNPa [9]. Using the AutoSNPa’s default settings, we performed shared autozygome detection among the patients and the rest of the family members that were used as healthy controls for the comparison. Among the detected ROH, a major block was identified toward the end of the long arm of chromosome 3 (Fig. 1b). This block was taken into consideration during the WES filtering as well and a homozygous variant was detected in (NM_014687:exon18:c.2624delC:p.A875fs) *RUBCN* (Fig. 1c). This variant, a single base deletion of cytosine in 2624th position, is previously reported in another Saudi family [1]; hence raising the possibility of being a founder mutation among Arab ethnic group or at least in Saudi population. Therefore we decided to utilize AutoSNPa findings as a reference point to investigate our hypothesis.

Previous study by Assoum et al. [1] indicated a shared region of homozygosity by descent on chromosomes 3q27.3-qter extending between rs719872 (189.4 Mb) and rs2363306 (199.3 Mb) in which RUBCN is located in addition to several other genes. This corresponds approximately 9.9 Mb region and contains the shared autozygosity detected in our family by AutoSNPa analysis (Fig. 1b). The analysis revealed distal (SNPs: rs35487798, chr3:197,152,191) and proximal (rs13094133, chr3:197,848,125) boundaries of the haplotype covering 695,934 bases. Indeed, our AutoSNPa is a much smaller region within the previously detected region in Assoum et al. study [1]. This is mainly because the previous study used 10 K SNP arrays that contains approximately 10,000 SNPs whereas we utilized much denser custom based axiom arrays comprising more than 600,000 informative SNPs. Hence using a denser array strategy we were able to narrow down the target autozygous region. Based on this new region, we calculated the genetic distance of the markers and calculated the age of the mutation as reported before [4, 10]. Mutation age analysis revealed the mutation occurred approximately 1550 years ago spanning about 62 generations.

## Discussion and conclusion

To date, only a single Saudi Arabian family with Salih ataxia (SCAR15, OMIM # 615705) has been documented [1, 2]. In the present communication, we validate the clinical phenotype of a second unrelated Saudi family harboring the same homozygous frameshift mutation (NM_014687:exon18:c.2624delC:p.A875fs) in *RUBCN* (*KIAA0226*) that has been described in the first family [2]. The original Saudi Arabian family had 3 sisters with unsteady gait apparent since learning to walk in two and around age 7 years in the third. Two developed epilepsy in infancy, which responded to treatment, and later manifested moderate intellectual disability (ID). Increased reflexes in the lower limbs were detected in two patients. Brain MRI was normal at ages 16, 9, and 8 years, respectively, but showed mild cerebellar atrophy and prominent folia in one patient at age 18 years. The two affected males (aged 6.5 and 17 years) belonging to the present second family also presented with unsteady gait apparent since learning to walk at 2.5 and 3 years, respectively. Both had cognitive impairment. The younger patient showed gait ataxia and normal reflexes, whereas the older patient had dysarthria, upper limb involvement, and increased reflexes in the lower limbs. MRI was normal at 2.5 years in the younger sibling (Patient 1) and at the age of 6 years in Patient 2. At the age of 16 years, the MRI revealed minimal superior vermian atrophy in the latter patient (Patient 2).

Similarities in the clinical phenotype in both families (Table 1) included delayed walking and speech acquisition, and the early onset of ataxia (since starting to walk). Also, ataxia is slowly progressive, manifesting with gait and limb ataxia, dysarthria (since starting to talk), and nystagmus or saccadic pursuit (in the second decade). Enhanced deep tendon jerks of the lower limbs were observed in Patient 2 (aged 17 years) and in two of three patients in the original family [1]. Plantar reflex was uniformly flexor in both families. Nevertheless, the remarkable difference between the two families is the development of epilepsy in infancy (that was responsive to treatment) in two of three patients in the original family, whereas none of the two patients in the present family had history of seizure disorder.

In comparison, other autosomal recessive cerebellar ataxias associated with epilepsy and ID can be differentiated by the associated presence of abnormalities in the peripheral nerves, basal ganglia, pyramidal tract, hearing or vision [11].

The first Saudi family with Salih ataxia (SCAR15) represents the first documentation of Rubicon’s effects on human health [1, 2, 12]. Truncated Rubicon was demonstrated to lose its ability to colocalize with Rab7 at late endosomes leading to defective endosomal trafficking [2]. Rubicon acts as a negative regulator of canonical autophagy and endosomal trafficking, plays a critical role in LC3-associated phagocytosis (a form of noncanonical autophagy [13]), and acts as a key modulator of viral replication and inflammatory response [12].

Both families have identical mutation in *RUBCN*, and further analysis revealed mutation age of approximately 1550 years spanning about 62 generations. Another founder mutation causing ataxia in the Arab population has recently been reported in patients from Oman with spinocerebellar ataxia with axonal neuropathy-1 (SCAN1; OMIM #607250) [14]. Identical homozygous variant in TDP1, the causative gene of SCAN1, has previously been documented in only a single family from Saudi Arabia in 2002 [15].

We validated the slowly progressive phenotype of Salih ataxia (SCAR15, OMIM # 615705) in a second Saudi family and its association with delayed motor development and impaired cognitive ability. Haplotype sharing attests to a common founder ancient RUBCN mutation in the Arab population.

## Data Availability

Data supporting our findings in this case report can be found in the Figs. 1, 2 and 3, and Table 1.

## Abbreviations

BAER: Brain Auditory Evoked Response
CBC: Complete Blood Count
CK: Creatine Kinase
F: Female
IQ: Intelligence Quotent
L: Left
M: Male
Mo: Months
MRI: Magnetic Resonance Imaging
NCS: Nerve Conduction Study
NICU: Neonatal Intensive Care Unit
R: Right
VEP: Visual Evoked Potentials
WES: Whole Exome Sequence
Y: Year
